# Magnesium sulphate for fetal neuroprotection: a cost-effectiveness analysis

**DOI:** 10.1186/1472-6963-13-527

**Published:** 2013-12-19

**Authors:** Celeste D Bickford, Laura A Magee, Craig Mitton, Marie Kruse, Anne R Synnes, Diane Sawchuck, Melanie Basso, Vyta M Senikas, Peter von Dadelszen

**Affiliations:** 1 School of Population and Public Health, Faculty of Medicine, University of British Columbia, Vancouver, Canada; 2 Department of Medicine, Division of Internal Medicine, University of British Columbia, Vancouver, Canada; 3 Danish Institute for Health Services Research, Copenhagen, Denmark; 4 Department of Pediatrics, Division of Neonatology, Children’s & Women’s Health Centre of British Columbia, Vancouver, Canada; 5 Department of Obstetrics and Gynecology, Division of Maternal Fetal Medicine, University of British Columbia, Vancouver, Canada; 6 Children’s & Women’s Health Centre of British Columbia, Perinatal Health Program, Vancouver, Canada; 7 Faculty of Medicine, McGill University, Montreal, Canada

**Keywords:** Magnesium sulphate, Fetal neuroprotection, Preterm birth, Cerebral palsy, Cost-effectiveness

## Abstract

**Background:**

The aim of this study was to assess the cost-effectiveness of administering magnesium sulphate to patients in whom preterm birth at < 32^+0^ weeks gestation is either *imminent* or *threatened* for the purpose of fetal neuroprotection*.*

**Methods:**

Multiple decision tree models and probabilistic sensitivity analyses were used to compare the administration of magnesium sulphate with the alternative of no treatment. Two separate cost perspectives were utilized in this series of analyses: a health system and a societal perspective. In addition, two separate measures of effectiveness were utilized: cases of cerebral palsy (CP) averted and quality-adjusted life years (QALYs).

**Results:**

From a health system and a societal perspective, respectively, a savings of $2,242 and $112,602 is obtained for each QALY gained and a savings of $30,942 and $1,554,198 is obtained for each case of CP averted when magnesium sulphate is administered to patients in whom preterm birth is *imminent*. From a health system perspective and a societal perspective, respectively, a cost of $2,083 is incurred and a savings of $108,277 is obtained for each QALY gained and a cost of $28,755 is incurred and a savings of $1,494,500 is obtained for each case of CP averted when magnesium sulphate is administered to patients in whom preterm birth is *threatened*.

**Conclusions:**

Administration of magnesium sulphate to patients in whom preterm birth is *imminent* is a dominant (i.e. cost-effective) strategy, no matter what cost perspective or measure of effectiveness is used. Administration of magnesium sulphate to patients in whom preterm birth is *threatened* is a dominant strategy from a societal perspective and is very likely to be cost-effective from a health system perspective.

## Background

Cerebral palsy (CP) is associated with substantial healthcare and societal costs, as well as a significant reduction in health-related quality of life for those with moderate to severe levels of disability [1-3]. It is estimated that preterm infants constitute up to 32% of all cases of CP, with the overall prevalence of CP being approximately 2 cases per 1,000 births [4]. Antenatal administration of magnesium sulphate for fetal neuroprotection has been shown to reduce the incidence of CP among preterm infants [5-11]. These findings have subsequently led to the publication of clinical practice guidelines on the use of magnesium sulphate for fetal neuroprotection in Canada and Australia, and endorsement of the use of magnesium sulphate for fetal neuroprotection by the American College of Obstetricians and Gynaecologists [12-14].

The main purpose of this paper is to present the results of a cost-effectiveness analysis on the use of magnesium sulphate for fetal neuroprotection. However, it is also the first paper to report the per-patient cost to administer magnesium sulphate antenatally specifically for the purpose of fetal neuroprotection and the first to present previously unpublished data on the lifetime cost of CP stratified by level of physical disability.

## Methods

### Model structure

**Figure 1:**
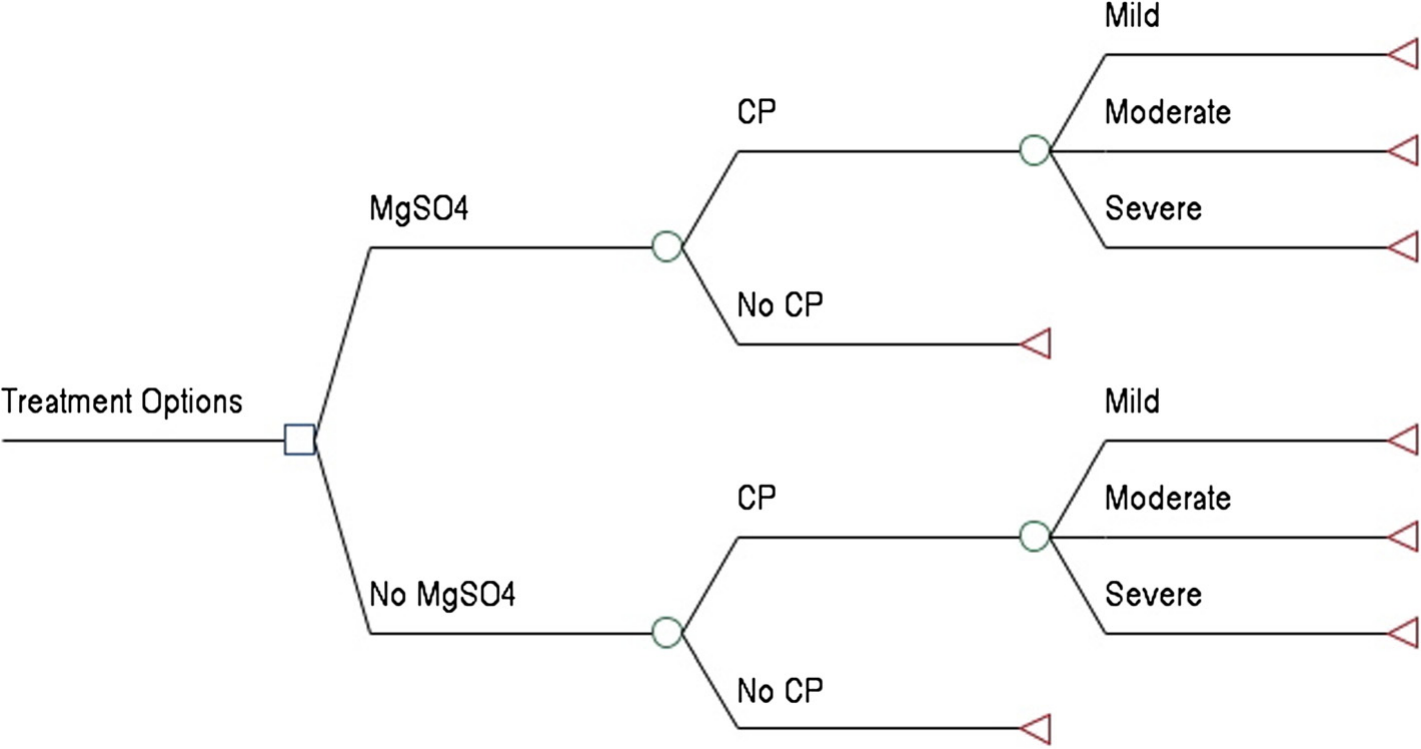
**Decision tree for the imminent preterm birth models.** The decision tree used to compare standard care (no treatment) with administration of magnesium sulphate to patients in whom preterm birth at < 32^+0^ weeks gestation is *imminent*.

**Figure 2:**
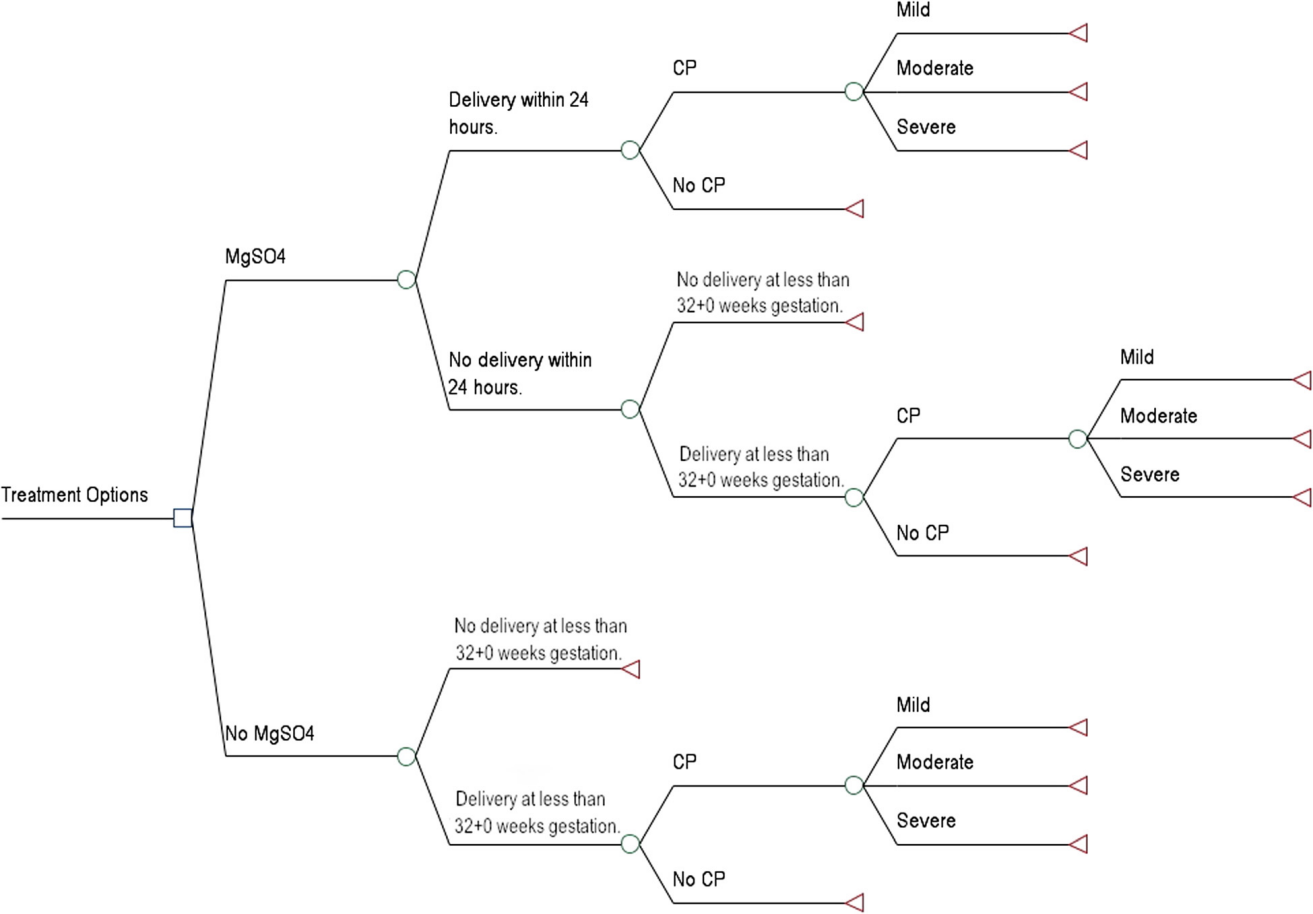
**Decision tree for the threatened preterm birth models.** The decision tree used to compare standard care (no treatment) with administration of magnesium sulphate to patients in whom preterm birth at < 32^+0^ weeks gestation is *threatened*.

Administration of magnesium sulphate was compared only with no treatment, as there are currently no other antenatal therapies used to prevent CP among preterm infants. The decision tree shown in Figure 1 was used to model the cost-effectiveness of administering magnesium sulphate to patients in whom preterm birth at < 32^+0^ weeks gestation is *imminent* (i.e. certain to occur within 24 hours). A separate decision tree (Figure 2) was constructed to model the cost-effectiveness of administering magnesium sulphate to patients in whom preterm birth at < 32^+0^ weeks gestation is *threatened* (i.e. could occur within 24 hours, but is not certain to), as there is no infallible way to identify all patients who will deliver within a given time period. Unpublished data gathered through the Canadian Perinatal Network suggest that only 24.4% of patients who present with indications such as preterm labour, antepartum hemorrhage, or premature prelabour rupture of the membranes deliver within 24 hours of admission to hospital [15]. As such, clinicians are likely to err by “overusing” magnesium sulphate among patients who present with *threatened* preterm birth. The decision tree shown in Figure 2 took into account: (i) that additional nursing costs would be incurred for patients in whom preterm birth at < 32^+0^ weeks gestation is *threatened*, as they would not otherwise need the one-on-one care required for administration of magnesium sulphate; (ii) that some would ultimately go on to deliver at more than 32^+0^ weeks gestation; and (iii) that some would be eligible to receive a second course of treatment with magnesium sulphate if they failed to deliver after the first course of treatment. It was assumed that re-treatment would only occur if there was a high degree of certainty that delivery was *imminent* and that all patients who received a second course of treatment would deliver at < 32^+0^ weeks gestation.

Maternal outcomes were excluded from the models, as there are only a few for which statistically significant differences have been found between women who had magnesium sulphate administered antenatally and those who did not [5-7]. These include transient hypotension, tachycardia, flushing, and nausea, which can all be reversed by discontinuing infusion of the drug. Note that the impact on the patient’s overall health utility is relatively small for each of these outcomes and the time period spent in each state of reduced health utility is brief, thereby making any difference in the quality-adjusted life years (QALYs) between the two groups negligible. The calculation of this difference would therefore not contribute meaningfully to this analysis.

CP was the only neonatal outcome included in the models, as it is the only outcome for which a statistically significant difference has been found between neonates who were antenatally exposed to magnesium sulphate for the purpose of fetal neuroprotection and those who were not [5-7]. CP has been stratified based on level of physical disability for the present analysis, with *mild* corresponding to levels I and II of the Gross Motor Function Classification System (GMFCS), *moderate* corresponding to level III, and *severe* corresponding to levels IV and V [16].

### Probabilities

The estimated probabilities for CP and each level of physical disability are shown in Table 1. Data from four RCTs were pooled to obtain these probabilities (Table 2) [8-11]. For the *threatened* preterm birth model (Figure 2), the probability of delivery within 24 hours of initiation of a single treatment with magnesium sulphate was estimated to be 0.2442 and the probability of re-treatment (given no delivery after an initial course of treatment) was estimated to be 0.6041 based on previously unpublished data from the Canadian Perinatal Network [15].

**Table 1:** **Estimated probability of CP among children born at < 32**^**+0**^ **weeks gestation and estimated probability of each level of physical disability given a diagnosis of CP**

	**MgSO**_**4**_	**No MgSO**_**4**_	**p-value**
CP	0.0524	0.0736	0.0144
Mild (GMFCS level I/II)	0.6364	0.6034	0.0552
Moderate (GMFCS level III)	0.2597	0.2069	0.0004
Severe (GMFCS level IV/V)	0.1039	0.1897	0.0001

**Table 2:** Data used to calculate the probability of CP

	**MgSO**_**4**_	**No MgSO**_**4**_	**Source**
Marrett	352	336	8
CP	22	30	
Mittendorf	30	29	9
CP	3	0	
Crowther	533	513	10
CP	36	42	
Mild (GMFCS level I/II)	21	21	^a^
Moderate (GMFCS level III)	12	15	^a^
Severe (GMFCS level IV/V)	3	6	^a^
Rouse	1030	1107	11
CP	41	74	
Mild (GMFCS level I/II)	28	49	^b^
Moderate (GMFCS level III)	8	9	^b^
Severe (GMFCS level IV/V)	5	16	^b^

Total number of patients assessed and number with each outcome in each of the four trials. Note that only data on patients to whom magnesium sulphate was administered for the specific purpose of fetal neuroprotection was included in the present analyses.

^a^Assumed with reference to the study protocol and detailed descriptions of characteristics for each GMFCS level for children under the age of four.

^b^D. Rouse, MD, written communication, March 2011.

### Life expectancies

Life expectancies at birth were estimated using both published median survival times and expert opinion (Table 3) [17,18]. It has been assumed that those with mild CP have a life expectancy at birth equal to that of the general population in Canada [19].

**Table 3:** Estimated life expectancies at birth for individuals with CP stratified by level of physical disability, as well as maximum and minimum values used in the sensitivity analyses

	**Estimated life expectancy (years)**	**Min**	**Max**
Mild (GMFCS level I/II)	81	71	91
Moderate (GMFCS level III)	66	56	76
Severe (GMFCS level IV/V)	25	20	30

### Health-related quality of life

The Health Utilities Index Mark 3 (HUI-3) is a validated, multi-attribute health classification system used to measure health-related quality of life [2,3]. HUI-3 utility scores are generated using scores for eight health attributes (vision, hearing, speech, ambulation, dexterity, emotion, cognition, and pain) [20]. Data from two studies were combined using weighted averages to obtain HUI-3 utility scores for individuals with CP (Table 4) [2,3]. Note that utility values were estimated using expert opinion for the age ranges for which no data were available. HUI-3 utility scores for individuals in the general population are shown in Table 5[21,22].

**Table 4:** Mean HUI-3 utility scores for individuals with CP stratified by level of physical disability

**Age (years)**	**Mild**		**Moderate**		**Severe**		**Source**
	**(GMFCS level I/II)**		**(GMFCS level III)**		**(GMFCS level IV/V)**		
	**Mean**	**SE**	**Mean**	**SE**	**Mean**	**SE**	
0-4	0.79	0.24	0.66	0.25	0.47	0.22	--
5-12	0.72	0.26	0.54	0.27	0.24	0.22	--
13-20	0.66	0.28	0.41	0.29	0.01	0.23	2,3
21-22	0.61	0.32	0.47	0.28	-0.01	0.22	--
23-32	0.57	0.35	0.53	0.27	-0.04	0.21	3
> 32	0.57	0.35	0.53	0.27	-0.04	0.21	--

**Table 5:** Mean HUI-3 utility scores for the general population

**Age (years)**	**General population**		**Source**
	**Mean**	**SE**	
0-12	0.92	0.11	--
13-15	0.90	0.15	21
16-37	0.85	0.18	21
38-54	0.83	0.01	22
55-64	0.77	0.02	22
65-74	0.80	0.01	22
75-89	0.75	0.01	22

### Cost to administer magnesium sulphate

All costs are presented in 2011 Canadian dollars [$1CAN = $1US = £0.62GBP]. The average cost (per 10,000 patients) to administer magnesium sulphate to patients in whom preterm birth at < 32^+0^ weeks gestation is *imminent* was estimated to be $57.08 per patient. Variation exists in dosing regimens, therefore this estimate assumed an administration protocol similar to those outlined in the Canadian and Australian clinical practice guidelines (i.e. a 4 gram intravenous loading dose followed by a 1 gram per hour maintenance infusion for a maximum of 24 hours) and took into consideration (1) the drug acquisition cost, (2) any laboratory costs incurred, and (3) the incremental cost of treating any adverse maternal or neonatal events associated with administering the drug [12,13]. No incremental nursing costs were added for monitoring patients in whom preterm birth at < 32^+0^ weeks gestation is *imminent*, as they would already be receiving one-on-one nursing care in a delivery suite.

A per patient drug acquisition cost of $34.35 was obtained from the pharmacy at an academic tertiary medical center in Vancouver, Canada. This included a pharmacy cost of $22.14 for one 1 liter bag of pre-mixed magnesium sulphate at a concentration of 40 mg/mL in normal saline (includes the drug and mixing costs), a cost of $1.37 each for three 1 liter bags of normal saline, a cost of $5.37 for one set of IV tubing, and a cost of $2.73 for one IV cannula (M. Tofan, Dipl Pharm, written communication, November 2011). Pre-mixing of the magnesium sulphate infusion was preferred to minimize the risk of dosage errors. It was assumed that each patient would already have an intravenous drip in place and that pumps would be readily available on the delivery unit.

Laboratory costs would only be incurred if there were evidence of maternal toxicity. It is anticipated that a maximum of 10% of patients would have one magnesium sulphate serum level measurement during the administration period, based on the proportion of women who discontinued infusion of the drug in the Magpie trial and who would have had serum levels measured if treated in a high income country [23]. The estimated cost for this measurement is $19.73 and is comprised of a $7.00 blood collection fee, a $5.00 handling fee, and a $7.73 laboratory fee (D. Sawchuck, PhD, written communication, August 2011).

It was assumed that the unlikely event of maternal respiratory depression would occur in a maximum 0.6% of patients, based on data from the Magpie trial [23]. It was further assumed that all of these patients would need to receive calcium gluconate and be intubated and ventilated for 24 hours. The estimated cost for this treatment is $2,884.30 and is comprised of a cost of $4.30 for a one gram dose of calcium gluconate (in a ten milliliter single use vial), a $280.00 airway management fee by an anesthesiologist, and $2,600.00 for 24 hours of critical care [24].

Excessive exposure to magnesium sulphate may result in neonatal hypotonia and respiratory depression [25,26]. It was assumed that the unlikely event of neonatal respiratory depression would occur in a maximum 0.6% of patients, based on the need for bag-mask ventilation in a sub-analysis of the BEAM trial [27]. This would require intubation and the need for level III NICU care for a period of 24 hours. To account for the maximum possible incremental cost, it was assumed that all neonates born at < 32^+0^ weeks gestation would otherwise require level II NICU care and would not be intubated. The estimated cost associated with this treatment is $576.03 and is comprised of an incremental cost of $126.03 for physician billing fees and an incremental cost of $450.00 for 24 hours of critical care in a level III NICU [28].

The average cost (per 10,000 patients) to administer magnesium sulphate to patients in whom preterm birth at < 32^+0^ weeks gestation is *threatened* was estimated to be $897.08 per patient. It was assumed that patients in whom preterm birth at < 32^+0^ weeks gestation is *threatened* would not already be receiving the one-on-one nursing care required for administration of magnesium sulphate. Therefore, the estimated cost to administer magnesium sulphate to this population included both the $57.08 administration cost outlined above and $840.00 in nursing costs to account for a 24 hour period of one-on-one nursing care (D. Sawchuck, PhD, written communication, August 2011).

### Lifetime cost of cerebral palsy

The lifetime costs of CP (Table 6) were estimated using the life expectancies shown in Table 3 and the health care, productivity, and social annual attributable cost inputs shown in Table 7. Detailed information on the methodology used to determine these annual attributable cost inputs has been outlined in a previous publication [1]. The health care costs consisted of all primary health care, hospital, and pharmaceutical costs. The productivity costs consisted of all costs associated with lost labour market productivity for adults with CP. The social costs consisted of all costs associated with specialized education, specialized housing, and lost labour market productivity for primary care providers of children with CP. Health care and productivity costs were quantified using patient-level data obtained from a Danish population-based register, while social costs were estimated using a combination of register data, previously published literature, and expert opinion.

**Table 6:** The undiscounted lifetime cost of CP stratified by level of physical disability

	**Health system perspective**	**Societal perspective**
Mild (GMFCS level I/II)	33,524	3,581,722
Moderate (GMFCS level III)	70,923	4,500,094
Severe (GMFCS level IV/V)	61,378	2,208,153

**Table 7:** The annual attributable health care, productivity, and social costs for children and adults with CP stratified by level of physical disability

	**Children ≤18 years**				**Adults > 18 years**			
	**Health**	**Productivity**	**Social**	**Total**	**Health**	**Productivity**	**Social**	**Total**
Mild (GMFCS level I/II)	1,279	0	51,146	**52,425**	146	16,991	23,905	**41,042**
Moderate (GMFCS level III)	2,895	0	77,179	**80,074**	331	25,653	36,072	**62,056**
Severe (GMFCS level IV/V)	3,100	0	87,278	**90,378**	355	28,992	40,792	**70,139**

All costs were originally determined in 2006 Danish Kroner (DKK). These values were converted to Canadian dollars (CAD) using the average Bank of Canada nominal exchange rate between January 1, 2002 and January 1, 2011 (1 CAD = 4.9494 DKK). Inflation was then adjusted for using Canadian Health and Personal Care Consumer Price Index (CPI) values for 2006 and 2011, which were 105.9 and 117.1 respectively. The formula used for currency conversion was: (DKK_2006_)*(1.0000 CAD)/(4.9494 DKK) = (CAD_2006_). The formula used to adjust for inflation was: (CAD_2006_)*(CPI_2011_)/(CPI_2006_) = (CAD_2011_).

### Analysis

The cost-effectiveness of antenatal administration of magnesium sulphate for fetal neuroprotection among preterm deliveries at < 32^+0^ weeks gestation as compared to no treatment were analysed from both a health system perspective and a societal perspective. Two measures of effectiveness were explored in separate analyses: (1) cases of CP averted and (2) QALYs. QALYs are a measure of effectiveness that take into account both the length of life and the level of health-related quality of life associated with a particular outcome [29,30]. Lifetime costs were used in all analyses.

Incremental cost-effectiveness ratios (ICERs) were calculated in the base-case analyses. ICERs give the additional cost per unit of effectiveness gained when one treatment alternative is compared to another [29,30]. Probabilistic sensitivity analyses were conducted using Monte Carlo simulation, producing incremental cost-effectiveness plots (ICEPs) and cost-effectiveness acceptability curves (CEACs). ICEPs illustrate the ICERs generated when model inputs are simultaneously varied in a series of analyses (e.g. 10,000 simulations). CEACs illustrate the probability of a treatment option being cost-effective across various willingness-to-pay values. Each willingness-to-pay value represents the maximum cost that a decision-maker is willing to pay for an additional unit of effectiveness [29,30].

The results of the Monte Carlo analyses are based on 10,000 simulations. Beta and Dirichlet distributions were used for probabilities, gamma distributions were used for costs, beta distributions were used for utility weights, and triangular distributions were used for life expectancies [29]. The standard errors for all costs were estimated to be 20% of their point estimates, to account for any uncertainty or regional variation in these values. The standard errors for all utility weights were taken from the literature (Tables 4 and 5). Minimum and maximum values for life expectancy estimates for individuals with CP were determined using expert opinion (Table 3). The life expectancy for the general population was not varied, as it was calculated using population-based data. In accordance with the World Health Organization’s guide to cost-effectiveness analyses, costs and QALYs were discounted at 3% annually for the base case analyses, but further sensitivity analyses were also conducted using a 6% annual discount rate for costs and a 0% annual discount rate for QALYs [30,31]. All analyses were carried out using TreeAge Pro 2012 (TreeAge Software, Inc, Williamstown, MA).

## Results

### *Imminent* preterm birth

A treatment option that is less costly and more effective than its alternative is always considered to be cost-effective and is referred to as a dominant strategy [30]. The point estimates listed in Tables 8 and 9 indicate that administration of magnesium sulphate is a dominant strategy compared to the alternative of no treatment no matter what measure of effectiveness or cost perspective is used. The results generated from a health system perspective indicate that a savings of $2,242 is obtained for each QALY gained and a savings of $30,942 is obtained for each case of CP averted. The results generated from a societal perspective indicate that a savings of $112,602 is obtained for each QALY gained and a savings of $1,554,198 is obtained for each case of CP averted.

**Table 8:** ICERs calculated for the imminent preterm birth models using cases of CP averted as the measure of effectiveness

**Analysis**	**Method**	**Cost**	**Δ cost**	**Cases w/o CP**	**Δ cases w/o CP**	**ICER**	**Status**
Health system perspective	MgSO_4_	$1,672	-$653	0.95	0.02	-$30,942	Dominant
	No MgSO_4_	$2,326		0.93			
Societal perspective	MgSO_4_	$85,822	-$32,808	0.95	0.02	-$1,554,198	Dominant
	No MgSO_4_	$118,630		0.93			

**Table 9:** ICERs calculated for the imminent preterm birth models using QALYs as the measure of effectiveness

**Analysis**	**Method**	**Cost**	**Δ cost**	**QALYs**	**Δ QALYs**	**ICER**	**Status**
Health system perspective	MgSO_4_	$1,672	-$653	26.6	0.3	-$2,242	Dominant
	No MgSO_4_	$2,326		26.3			
Societal perspective	MgSO_4_	$85,822	-$32,808	26.6	0.3	-$112,602	Dominant
	No MgSO_4_	$118,630		26.3			

**Figure 3:**
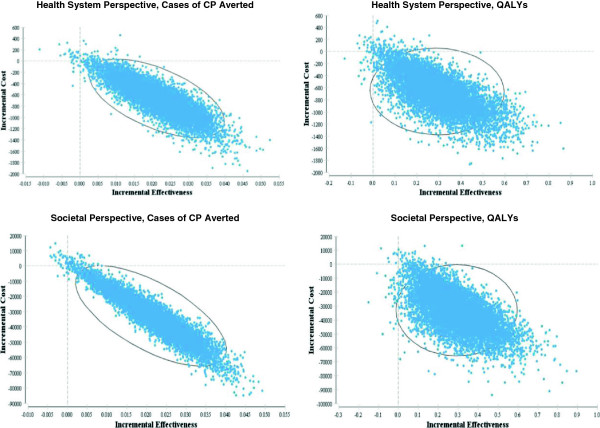
**ICEPs for the imminent preterm birth models.** The black ellipses represent 95% confidence intervals.

**Figure 4:**
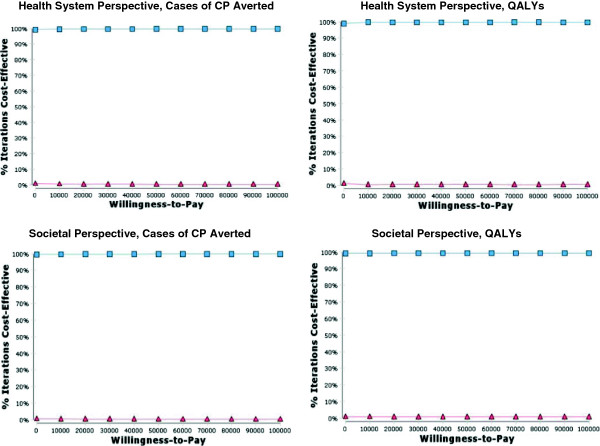
**CEACs for the imminent preterm birth models.** The blue line depicts the probability of magnesium sulphate being cost-effective across all willingness-to-pay values up to $100,000. The red line depicts the probability of the alternative (no treatment) being cost effective across all willingness-to-pay values up to $100,000.

The ICEPs generated through the probabilistic sensitivity analysis illustrate that when all model inputs are varied, the majority of the computed ICERs fall into the quadrant of the incremental cost-effectiveness plane which indicates that administering magnesium sulphate is a dominant strategy compared to the alternative of no treatment (Figure 3). The CEACs indicate that the probability of administration of magnesium sulphate being cost-effective is over 99% for all willingness-to-pay values up to $100,000, no matter what measure of effectiveness or cost perspective is used (Figure 4).

### *Threatened* preterm birth

The point estimates listed in Tables 10 and 11 indicate that administration of magnesium sulphate is a dominant strategy compared to the alternative of no treatment only when a societal perspective is used. When a health system perspective is used, it is both more effective and more costly than the alternative of no treatment. The results generated from a societal perspective indicate that a savings of $108,277 is obtained for each QALY gained and a savings of $1,494,500 is obtained for each case of CP averted. The results generated from a health system perspective indicate that a cost of $2,083 is incurred for each QALY gained and a cost of $28,755 is incurred for each case of CP averted.

**Table 10:** ICERs calculated for the threatened preterm birth models using cases of CP averted as the measure of effectiveness

**Analysis**	**Method**	**Cost**	**Δ cost**	**Cases w/o CP**	**Δ cases w/o CP**	**ICER**	**Status**
Health system perspective	MgSO_4_	$2,055	$425	0.96	0.01	$28,755	^a^
	No MgSO_4_	$1,630		0.95			
Societal perspective	MgSO_4_	$61,028	-$22,109	0.96	0.01	-$1,494,500	Dominant
	No MgSO_4_	$83,137		0.95			

^a^Cost-effectiveness is dependent on the willingness-to-pay threshold of the decision-maker.

**Table 11:** ICERs calculated for the threatened preterm birth models using QALYs as the measure of effectiveness

**Analysis**	**Method**	**Cost**	**Δ cost**	**QALYs**	**Δ QALYs**	**ICER**	**Status**
Health system perspective	MgSO_4_	$2,055	$425	26.7	0.2	$2,083	^a^
	No MgSO_4_	$1,630		26.5			
Societal perspective	MgSO_4_	$61,028	-$22,109	26.7	0.2	-$108,277	Dominant
	No MgSO_4_	$83,137		26.5			

^a^Cost-effectiveness is dependent on the willingness-to-pay threshold of the decision-maker.

**Figure 5:**
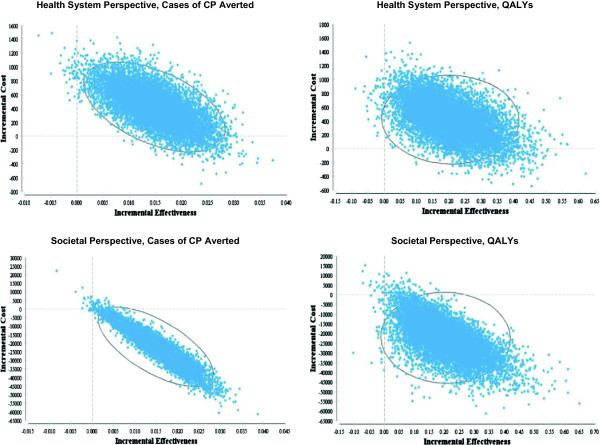
**ICEPs for the threatened preterm birth models.** The black ellipses represent 95% confidence intervals.

**Figure 6:**
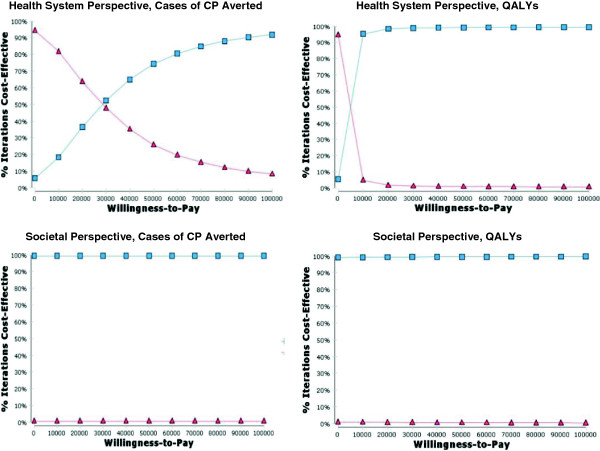
**CEACs for the threatened preterm birth models.** The blue line depicts the probability of magnesium sulphate being cost-effective across all willingness-to-pay values up to $100,000. The red line depicts the probability of the alternative (no treatment) being cost effective across all willingness-to-pay values up to $100,000.

The ICEPs generated through the probabilistic sensitivity analysis illustrate that when all model inputs are varied, the majority of the ICERs computed from a societal perspective fall into the quadrant of the incremental cost-effectiveness plane which indicates that administration of magnesium sulphate is a dominant strategy compared to the alternative of no treatment. Conversely, the majority of the ICERs computed from a health system perspective fall into the quadrant of the incremental cost-effectiveness plane which indicates that that administration of magnesium sulphate is both more costly and more effective than the alternative of no treatment (Figure 5). The CEACs generated using a societal perspective indicate that the probability of administration of magnesium sulphate being cost-effective is over 99% for all willingness-to-pay values up to $100,000, no matter what measure of effectiveness is used. The CEACs generated using a health system perspective indicate much lower probabilities of cost-effectiveness (Figure 6).

## Discussion

### Main findings

Administration of magnesium sulphate to patients in whom preterm birth at < 32^+0^ weeks gestation is *imminent* is a dominant (i.e. cost-effective) strategy compared to the alternative of no treatment, no matter what measure of effectiveness or cost perspective is used. Administration of magnesium sulphate to patients in whom preterm birth at < 32^+0^ weeks gestation is *threatened* is a dominant strategy compared to the alternative of no treatment from only a societal perspective. When a health system perspective is used its cost-effectiveness is dependent on the decision-makers’ willingness-to-pay for an additional QALY or case of CP averted. The National Institute for Health and Clinical Excellence suggests that society’s willingness-to-pay threshold for new health technologies is approximately $30,000/QALY, making anything less costly a cost-effective option from the viewpoint of society as a whole [32]. Administration of magnesium sulphate to patients in whom preterm birth at < 32^+0^ weeks gestation is *threatened* has a very high probability of being cost-effective at all willingness-to-pay values over $10,000/QALY, no matter what cost perspective is used. Therefore, it is very likely to be a cost-effective option from the viewpoint of society as a whole even though it is not a dominant strategy from all cost perspectives.

### Strengths and limitations

This is the first study in which the cost to administer magnesium sulphate specifically for fetal neuroprotection has been estimated (previous studies, including the Cahill *et al.* cost-effectiveness analysis, have only reported/utilized the cost to administer magnesium sulphate for tocolysis or preeclampsia prophylaxis). It is also the first study to present detailed data on the lifetime costs of CP by level of physical disability. The use of these data in the present analysis is important because the extent of care required for individuals with different levels of disability varies considerably. It is also important because the lifetime cost of CP is a key driver of the cost-effectiveness of administration of magnesium sulphate.

The analyses included models for both *imminent* and *threatened* preterm birth because there is no infallible way to predict which patients will deliver imminently. As such, clinicians are likely to err by “overusing” magnesium sulphate among patients who present with what appears to be *imminent* preterm birth, but is in fact *threatened* preterm birth. The *threatened* preterm birth model included important information on additional resources required to administer magnesium sulphate to those who would be likely to receive it in clinical practice. However, note that the assumption that a second course of treatment with magnesium sulphate would occur only in the case of *imminent* preterm birth may not always reflect clinical practice.

The estimation of model inputs is a common limitation of decision analytic models. For the present analysis, many of the utility weights for individuals with CP were estimated due to a paucity of published information on the health-related quality of life of this population. Uncertainty also remains around the probability inputs, given that they were obtained using aggregated data rather than data from a single randomized controlled trial [33]. In this analysis the impact that such estimates have on the precision of the model has been minimized through the use of a robust probabilistic sensitivity analysis, which allows the effect of uncertainty across all the parameters to be considered simultaneously.

Another common limitation of decision analytic models is the uncertainty that surrounds the structure of the model itself. For example, it should be noted that the classification of CP as being mild, moderate, or severe requires broad generalizations and that this is only one of many classification systems that could have been utilized. In addition, perinatal death was excluded from the model, as the authors feel that there is not enough evidence at this time to indicate whether there is a difference in perinatal death between the two groups or what the magnitude of this difference may be. However, it should be noted that the assumption of no effect on mortality has been recently disputed in a meta-analysis of observational studies, which found that the use of magnesium sulphate was associated with a decrease in both CP and perinatal mortality [34]. With respect to these findings, if a randomized controlled trial were ever to show that antenatal administration of magnesium sulphate decreased the probability of perinatal death, then the cost-effectiveness of administering magnesium sulphate for fetal neuroprotection would only be improved.

### Comparison to other studies

One other cost-effectiveness analysis on the use of magnesium sulphate for fetal neuroprotection has been identified [35]. The results obtained in this study are consistent with the findings of our analysis in that administration of magnesium sulphate for fetal neuroprotection was found to be less costly and more effective than the alternative of no treatment from a societal perspective. However, our probabilistic sensitivity analysis produced much higher probabilities of magnesium sulphate being cost-effective. An explanation for this difference is that the cost of CP used in the Cahill *et al.* study was likely underestimated. They did not take into account the severity of CP and their estimate of the lifetime cost of CP was taken from a single study, whereas our analysis used previously unpublished, population-based data on the lifetime cost of CP stratified by level of physical disability. Furthermore, the life expectancies and utility values utilized in their analysis were likely overestimated. We have therefore built on the Cahill *et al.* study by inserting more precise estimates into our model, in addition to using several cost perspectives and measures of effectiveness and investigating the cost-effectiveness of *imminent* and *threatened* preterm birth separately.

## Conclusions

Administration of magnesium sulphate to patients in whom preterm birth is *imminent* is a dominant (i.e. cost-effective) strategy, no matter what measure of effectiveness or cost perspective is used. Administration of magnesium sulphate to patients in whom preterm birth is *threatened* is a dominant strategy from a societal perspective and is very likely to be cost-effective from a health system perspective. Clinicians and administrators should interpret the findings of this study as providing reassurance that if a diagnosis of *imminent* preterm birth at < 32^+0^ weeks gestation is made and magnesium sulphate is administered, but the patient does not deliver, this “overuse” of magnesium sulphate is still likely to have been cost-effective.

## Competing interests

The authors declare that they have no competing interests.

## Authors’ contributions

CB contributed to the literature review, study design, and analysis. CB drafted the first version of the manuscript and led the submission process. LM initiated the collaborative project, as well as contributing to the study design and analysis and obtaining costing data. CM, MK, AS, DS, MB, VS, and PvD contributed to the study design and analysis and obtained costing data. All authors contributed to the interpretation of the study findings and the revision and approval of the final manuscript.

## Pre-publication history

The pre-publication history for this paper can be accessed here:

http://www.biomedcentral.com/1472-6963/13/527/prepub
